# Body Composition and Dietary Intake Profiles of Elite Iranian Swimmers and Water Polo Athletes

**DOI:** 10.3390/nu16152393

**Published:** 2024-07-24

**Authors:** Mohammad Hossein Samanipour, Shahzad Mohammadian, Juan Del Coso, Omid Salehian, Fatemeh Khodakhah Jeddi, Mehdi Khosravi, José M. González-Ravé, Halil İbrahim Ceylan, Hongyou Liu, Sidney Abou Sawan, Ralf Jäger

**Affiliations:** 1 Department of Sport Science, Imam Khomeini International University, Qazvin 34148-96818, Iran; 2 Department of Sport Nutrition, National Paralympic Committee (NPC), Tehran 19956-13114, Iran; 3 Centre for Sport Studies, Universidad Rey Juan Carlos, 28922 Madrid, Spain; juan.delcoso@urjc.es; 4 Department of Sport Nutrition and Fitness, Applied and Science University, Tehran 13114-16846, Iran; 5 Research Center Faculty of Nutrition, Tabriz University of Medical Science, Tabriz 51656-87386, Iran; 6 Department of Human Sciences, North Tehran Branch, Islamic Azad University, Tehran 14778-93855, Iran; 7 Sports Training Laboratory, Faculty of Sports Sciences, University of Castilla La Mancha, 13071 Toledo, Spain; josemaria.gonzalez@uclm.es; 8 Physical Education and Sports Teaching Department, Faculty of Sports Sciences, Atatürk University, 25030 Erzurum, Turkey; halil.ibrahimceylan60@gmail.com; 9 School of Physical Education & Sports Science, South China Normal University, Guangzhou 510631, China; szu.youyou@hotmail.com; 10 Iovate Health Sciences International, Oakville, ON L6M 0H4, Canada; sidney.abousawan@iovate.com; 11 Increnovo LLC, Whitefish Bay, WI 53217, USA

**Keywords:** body composition, energy demands, nutrients, human body, aquatic sports

## Abstract

Background: This study aimed to conduct a detailed and comparative analysis of body composition and dietary habits in elite swimming and water polo athletes. Through the examination of these key parameters, this study seeks to compare the dietary intake of these two distinct aquatic sports disciplines. Methods: A total of 10 top-level swimmers and 13 water polo athletes participated in anthropometric and body composition assessments, as well as a detailed analysis of nutritional intake. To compare the two groups, an independent samples *t*-test was used, and variance homogeneity was checked using Levene’s test. The effect size of the group differences was evaluated using Hedges’ g. Results: Water polo athletes showed significantly greater height (189.4 ± 2.9 vs. 186.5 ± 2.0 cm, *p* = 0.013), body mass index (24.3 ± 1.4 vs. 22.1 ± 0.5 kg/m^2^, *p* < 0.001), fat-free mass (62.9 ± 1.4 vs. 61.1 ± 1.38 kg, *p* < 0.001), skeletal muscle mass (47.1 ± 1.3 vs. 43.9 ± 1.6 kg, *p* < 0.001), and overall weight (86.9 ± 6.9 vs. 76.7 ± 2.2 kg, *p* < 0.001) in comparison to swimmers. Swimmers consumed greater amounts of mean daily energy (60.0 ± 1.0 vs. 39.0 ± 1.0 kcal/kg, *p* < 0.001), carbohydrate (7.8 ± 0.3 vs. 4.4 ± 0.5 g/kg, *p* < 0.001), protein (1.7 ± 0.5 vs. 1.4 ± 0.5 g/kg, *p* < 0.001), and fat (2.4 ± 0.5 vs. 1.7 ± 0.5 g/kg, *p* < 0.001) compared to water polo athletes. Conclusion: Our findings highlight the need for differentiated targeted nutritional interventions to enhance athletic performance in different types of water sports. Compared to water polo athletes, swimmers consumed significantly higher amounts of calories, matching their increased calorie demand from their specific training regime. However, this is an observational study and the differential needs of energy and macronutrients in water sports should be confirmed by studies with energy expenditure measurements.

## 1. Introduction

Aquatics encompass a group of sports that take place in swimming pools, lakes, or oceans. These sports, including swimming, water polo, diving, and synchronized swimming [1], share a common requirement: high levels of muscle strength and power to overcome unique challenges. While all aquatic sports share this fundamental requirement, they differ significantly in terms of rules and regulations and physical and nutritional demands. Swimming, being the most popular aquatic sport, involves races undertaken by individuals or teams, spanning various distances and employing different types of swimming strokes [2]. Swimmers exclusively rely on their bodily propulsive forces to move through water, thus highlighting the crucial role of strength and power. Additionally, the significance of aerobic endurance varies depending on factors such as the swimming style, intensity, and distance covered [3,4,5]. In swimming, the interplay between strength and technique plays a pivotal role, as swimmers must contend with hydrodynamic drag—a resistance that increases proportionally with swimming velocity, following the square law [3,4,5]. Conquering this drag necessitates a combination of well-developed strength and impeccable stroke technique, as swimmers strive to minimize resistance and optimize their performance in the water. It is worth noting that different swimming strokes require distinct techniques and emphasize various muscle groups, further accentuating the complexity of the sport.

Swimming has been a fundamental discipline in the Olympic Games since 1896, being a sport that is consistently featured in every Summer Games. Competitive swimming involves races dedicated to the four main strokes: freestyle, backstroke, butterfly, and breaststroke, either individually or in a medley format. The duration of swimming events can range from 20 s to 16 min, covering distances from 50 m to 1500 m, showcasing the sport’s intricate technical nature that requires a blend of power and endurance supported by varying anaerobic and aerobic energy systems. Additionally, the inclusion of relays in swimming competitions introduces a team dynamic to the overall format. The changing energy needs of a swimmer chiefly reflect his or her training/racing volume, growth or goals to alter physique, competitors, and other lifestyle activities. Typical values for the self-reported energy intakes of male and female swimmers are 3600–4800 kcal/day and1,900–2600 kcal/day, respectively [6]. Swimming has been linked anecdotally with heightened appetite and a greater risk of excessive eating in comparison to other physical activities; this phenomenon may be attributed to the decreased thermoregulatory stress resulting from the dissipation of heat in a cooler aquatic setting. In fact, research has demonstrated that a one-hour session of running in cold temperatures (10 °C) can lead to an increase in spontaneous food consumption and a decrease in satiety when contrasted with exercising in a milder, temperate environment (20 °C). On the contrary, engaging in the same exercise routine in warmer conditions (30 °C) has been found to diminish feelings of hunger and the desire for future food consumption [7].

Water polo, a distinct and competitive team sport, amalgamates diverse physical attributes such as swimming speed, agility, strength, endurance, and power. This sport requires tactical acumen and proficiency in technical skills, particularly ball control [8,9]. Water polo games consist of four quarters, each lasting 8 min plus potential overtime, resulting in total game times of 60–70 min. Notably, water polo exhibits a unique combination of acyclic elements reminiscent of conventional ball games, juxtaposed with cyclic components akin to swimming, with particular emphasis on performance and stroke biomechanics [9,10].

The nutritional needs of athletes are primarily determined by the specific demands of their sport and the overall goal to optimize athletic performance [11]. Optimal nutritional practices play a crucial role in competition and practice performance [12]. Optimizing subsequent recovery allows athletes to train harder and more frequently, resulting in faster training adaptations. Hence, athletes are advised to adhere to an optimal daily intake of essential nutrients, including proteins (PROs), carbohydrates (CHOs), and fats, adjusted to their personalized training volume and intensity typical for their respective sports. For instance, in swimming, the recommended CHO intake typically falls within the range of 6–12 g/kg/day, while protein consumption is advised to be around 2 g/kg/day [10,12,13]. Following exhaustive, high-intensity training, muscle glycogen stores deplete and adequate carbohydrate intake is needed to restore muscle glycogen stores for optimal performance. For swimmers engaged in moderate training, 6 g/kg/day of carbohydrate intake is sufficient to adequately replenish glycogen stores and the consumption of larger carbohydrate quantities (12 g/kg/day) did not result in any additional performance benefits [14]. Swimmers should consume 6–8 g/kg/day of carbohydrates on training days with high volume and low intensity or very low volume and high intensity. On days of moderate or high volume with high intensity, carbohydrate intake should be in the range of 10–12 g/kg/day [13,15]. Furthermore, fat intake should constitute to 20–25% of the daily caloric intake [11]. Athletic nutritional intake is aimed to provide all essential nutrients in optimal quantities from high quality food sources while avoiding superfluous ingestion of food items and preserving the athlete’s overall health status [12]. The nutritional status of athletes also exerts a discernible influence on anthropometric and body composition characteristics, which are contingent upon maintaining a correct energy balance [16]. Consequently, continuous monitoring of body composition and nutritional status assumes paramount importance in optimizing sports performance for swimmers and water polo athletes. Monitoring is also needed to identify and address potential nutritional deficiencies [10].

The primary objective of this study is to conduct a detailed and comparative analysis of body composition and dietary habits in elite swimming and water polo athletes. Through the examination of these key parameters, the study seeks to compare the dietary intake of these two distinct aquatic sports disciplines.

## 2. Methods

### 2.1. Study Design

During an eight-week training camp leading up to the 2018 Asian Games in Jakarta and Palembang, a cross-sectional study was conducted in elite athletes. The study period aligned with the competitive training phase intended to achieve peak performance. Throughout the duration of the training camp, all participating athletes took part in identical sessions involving estimated food intake and anthropometric measurements. Nutrition habits were assessed over three weekends to ensure accuracy. Anthropometric assessments and food intake recalls were repeated upon entry and conclusion of the training camp. This study was approved by the Sport Science Research Institute of Iran, Tehran, Iran (IR.SSRC.REC.1399.062, 15 March 2018).

### 2.2. Participants

**Figure 1:**
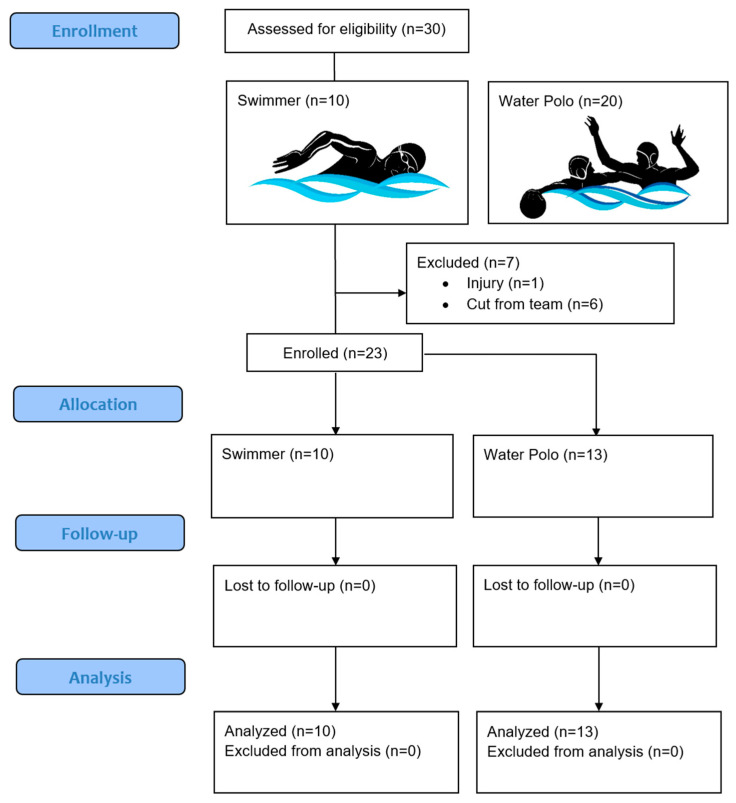
CONSORT flow diagram.

Ten elite male swimmers and 20 water polo players from the Iranian National Olympic Team were assessed for eligibility to participate in this study. One water polo player was injured and six were cut from the team prior to enrollment. Ten swimmers (22.6 ± 2.1 years) and 13 elite male water polo athletes (22.1 ± 2.5 years) were enrolled after signing a written consent to participate in this study (see Figure 1).

Swimmers had an average training experience of 10.6 ± 3.3 years, winning silver or bronze medals at global, Asian, or national competitions. The water polo athletes placed 5th in the Asian Championship and had an average training experience of 11.2 ± 2.5 years. To enroll in this study, athletes needed to complete 6 weeks of training with standardized training volumes focusing on high-intensity interval training. Athletes with a current injury or participating in additional aquatic sports (e.g., diving, triathlon) were excluded from the study. All athletes participating in this study were actively engaged in a systematic preparation training regimen, which encompassed four training macrocycles annually throughout the season, aimed at adequately preparing them for the Asian games. For swimmers, their average microcycle consisted of 7 sessions of 2 h each, conducted in the pool and focused on training for distances of 50 m and 200 m. Additionally, they underwent 4 land-based training sessions of 1 h each per week, up until one week before an international tournament. Water polo athletes, on the other hand, followed a training routine that comprised 6 sessions of 2 h each, conducted in the pool, along with 4 land-based training sessions of 1.5 h each per week. These land-based sessions included strength training and aerobic exercises, specifically designed to enhance their performance in water-polo. This research adhered strictly to the Code of Ethics of the World Anti-Doping Agency (WADA) to ensure the ethical conduct and integrity of the study. Prior to any assessments, all participants were provided with detailed information regarding the study procedures, and their informed consent was obtained in writing.

### 2.3. Body Composition and Anthropometric Measurements

Participants’ body weight (BW) and height were measured using a calibrated scale (SECA 769, Hamburg, Germany) and a stadiometer (SECA 242, Hamburg, Germany), respectively. To ensure accuracy, measurements were taken while participants were wearing light clothing and without shoes in the morning following an overnight fast. Body composition (BC) was measured using the InBody 720 (Biospace Co, Ltd., Seoul, Republic of Korea) device, which has a high test–retest reliability, with an Intraclass Correlation Coefficient of 0.9995 [17,18,19,20]. Body composition variables included fat mass (FM), fat-free mass (FFM), body fat percentage (%BF), and skeletal muscle mass (SMM). Additionally, body mass index (BMI) was calculated.

### 2.4. Nutrient Analysis

To ensure accuracy and minimize potential errors in dietary assessment, athletes were requested to complete a validated food frequency questionnaire (FFQ) over a span of three consecutive days [21]. The training intensity was above 85–95% maximum heart rate (HR_max_) in the 5 weeks before competition. Intensity of effort was monitored via a chest belt (Polar H10, Kempele, Finland) during training by practitioners and trainers. Moreover, all athletes performed high-intensity interval training (HIIT) for about 1.5 h (1 session) per week. Participants consistently underwent resistance training at an intensity of 70–80% of one-repetition maximum (1RM) for the upper and lower limbs for three sessions per week. In addition, they performed plyometric and resistance-band training for about 30 min during HIIT sessions. The intensity of effort was at the typical normal training of the subjects. Throughout this period, participants were advised to maintain their usual dietary habits. To further enhance the precision of dietary records, all subjects received a food album containing a comprehensive list of native Iranian cultural foods to facilitate accurate recording of food consumption. Researchers also assisted in the calculation of serving sizes and portions from the athletes’ FFQ responses. Subsequently, all collected dietary data were entered into the nutritional software NutriBase (version 7.18, CyberSoft, Incorporated, Phoenix, AZ, USA) to calculate macro- and micronutrient intake based on the participants’ body mass and daily consumption. The athletes completed the FFQ while resting in a recumbent position on a stretcher, ensuring their comfort and readiness to provide detailed and accurate dietary information. Data collection was performed on training day two weeks before competition. The FFQ captured comprehensive data concerning calorie intake, as well as consumption of CHO, PRO, fats (including monounsaturated fatty acids (MUFAs) and polyunsaturated fatty acids (PUFAs)), cholesterol, dietary fiber, vitamins (A, B group, C), and minerals (sodium, potassium, calcium, phosphorus, iron, and selenium).

### 2.5. Statistical Analysis

Data normality for each variable was assessed using the Shapiro–Wilk test. After confirming the normal distribution of the data, means, standard deviations (SD), and 95% confidence intervals (CIs) were calculated for the different variables. To compare the two groups, an independent samples Student’s *t*-test was employed. Before conducting the *t*-test, the assumption of variance homogeneity was tested using Levene’s test. To gauge the effect size of the group differences, Hedges’ g was computed [22]. The interpretation of effect size followed the criteria as follows: 0–0.19 trivial, 0.2–0.59 small, 0.6–1.19 moderate, 1.2–1.99 large, 2.0–3.99 very large, ≥4.0 extremely large [23]. For statistical analyses and data presentation, SPSS version 20.0 and GraphPad Prism version 8.0 were utilized. A significance level of alpha ≤ 0.05 was established as the threshold for determining statistical significance in this study. Data are presented as means ± SD.

## 3. Results

Table 1 presents the age and body characteristics of both swimmers and water polo athletes. Notably, water polo athletes demonstrated significantly higher BW and height compared to swimmers (*p* < 0.05), resulting in a higher BMI for the water polo group (*p* < 0.001). Moreover, water polo athletes exhibited greater SMM (*p* < 0.001) and FFM (*p* = 0.008) in comparison to swimmers. Conversely, swimmers displayed higher %BF and FM compared to WP athletes (*p* < 0.001).

**Figure 2:**
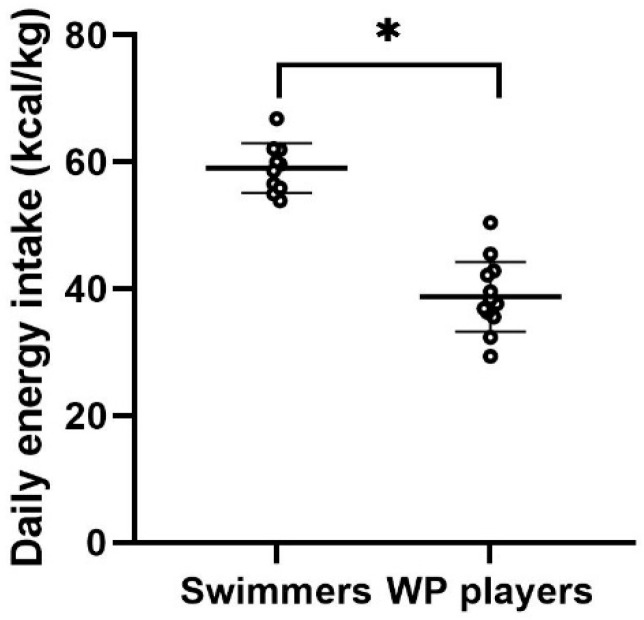
Mean ± SD and individual data for daily energy intake (kcal/kg) for swimmers and WP athletes; * *p* < 0.001.

Figure 2 displays both group and individual data of total energy intake (kcal/kg) for the swimmers and water polo athletes. Notably, swimmers exhibited a significantly higher energy intake compared to water polo athletes (t = 9.86; *p* < 0.001, ES = 4.1).

**Figure 3:**
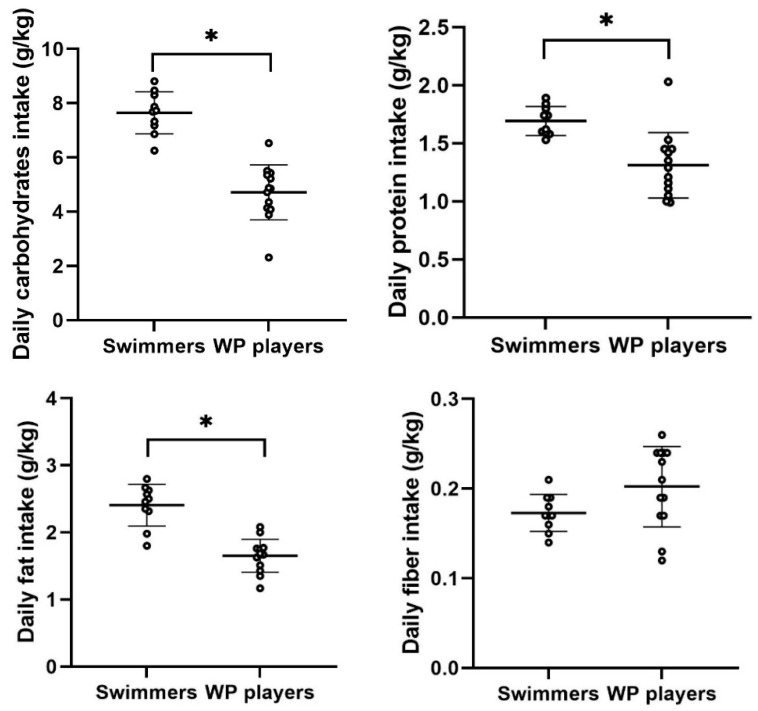
Mean ± SD and individual data for daily carbohydrate (* *p* < 0.001), protein (* *p* = 0.001), fat (* *p* < 0.001), and fiber relative to BW.

Figure 3 illustrates both group and individual data of daily macronutrient intake, namely CHO, PRO, and fat, as well as fiber intake relative to BW for both swimmers and water polo athletes. Swimmers exhibited significantly higher daily intake of CHO (t = 7.57; *p* < 0.001, ES = 3.2), PRO (t = 3.97; *p* = 0.001, ES = 1.60), and fat (t = 6.50; *p* < 0.001, ES = 2.75) compared to water polo athletes. However, there was no statistically significant difference in daily fiber intake between groups (t = −1.86; *p* = 0.076, ES = 0.91).

Water polo athletes demonstrated higher consumption of certain micronutrients compared to swimmers. Specifically, water polo athletes had higher intake of vitamins B3 and B12, selenium (Se), phosphorus (P), and sodium (Na^+^). Conversely, swimmers exhibited higher consumption of certain micronutrients compared to water polo athletes. Specifically, swimmers had higher intake of vitamins C and A (see Table 2).

## 4. Discussion

We conducted a detailed and comparative analysis of body composition and dietary habits in elite swimming and water polo athletes. Through the examination of these key parameters, the study compared the dietary intake of these two distinct aquatic sports disciplines. Water polo athletes were taller and heavier than swimmers, as indicated by their higher BMI and BW. Additionally, water polo athletes exhibited higher levels of SMM and FFM compared to swimmers. In contrast, swimmers demonstrated higher daily energy intake, as well as higher intake of CHO, PRO, and fat, after standardization for BW. These findings emphasize the importance of recognizing the distinct physiological and nutritional profiles of athletes in different aquatic sports disciplines.

Our findings partially confirmed previous research, which reported differences in BC between elite swimmers and water polo athletes [24]. These studies have consistently found that water polo athletes tend to be heavier and have higher body fat levels compared to elite swimmers. With 11.3% body fat, the swimmers in our study had similar body fat percentages compared to competitive Greek swimmers (11.8%) [25], international-level swimmers from eight different countries (9.9%) [26], and collegiate U.S. swimmers (14.1%) [27]. The body fat percentage of the water polo players in our study was not only lower than the ones of the swimmers, but they were also lower compared to elite Italian water polo athletes (19.1%) [28,29,30]. The higher energy intake in swimmers in our study compared to water polo athletes is a potential reason for the observed higher fat percentage.

A comparative study on water polo athletes over a period from 1980 to 1995 highlighted interesting trends in body measures, revealing changes in height, limb length, waist circumference, shoulder breadth, and muscle-to-fat-mass ratio over time [25]. The importance of the body structure characteristics that strongly contribute to the contractile potential of swimmers, regardless of gender, are determined by SMM, FFM, and the ratio of muscle and fat. All of these variables are correlated to strength and power production in sprint swimmers [26].

These changes were accompanied by higher body density and a lack of significant differences in BW. The authors speculated that these observed changes might be related to various factors, including ethnicity, culture, training load and experience, and sport-specific morphology. The present study focused on Iranian athletes, which may contribute to some unique characteristics observed in our sample. A study by Frits et al. [31] shows two significantly different clusters based on Hungarian male WP athletes’ anthropometric and body composition characteristics. Cluster I included goalkeepers and wingers, while Cluster II consisted of centers, shooters, and defenders. Cluster I athletes were 5 cm shorter on average, while their mean body weight, SMM, and FM were lower by 19 kg, 7 kg, and 7 kg, respectively, compared to the height, body weight, SMM, and FM of athletes classified in cluster II, while in our study swimmers had more FM than WP athletes. Furthermore, position-specific physical and morphological attributes may also play a role in differentiating body composition among water polo athletes [31,32]. For example, BC differences have been observed between water polo athletes playing at the center or back positions, with center athletes being heavier and having higher body fat levels and perimeter athletes being smaller and leaner [31,32]. The present study did not have sufficient statistical power to perform stratification according to the playing position. However, the consideration of position-specific characteristics in future research can offer valuable insights into the role of body composition in different roles within the water polo team.

The differences in dietary intake observed between elite swimmers and water polo athletes in the present study can be explained by considering the unique physiological demands of aquatic sports disciplines, especially in the case of water polo. Water polo athletes engage in a wide range of activities during match play, including swimming at various intensities, wrestling with opponents, lunging in offense and defense, treading water using the eggbeater kick, and performing passing, receiving, and shooting actions [10]. These activities require a considerable amount of energy and various macronutrients to support the athletes’ performance. During a water polo match, athletes typically have a total playing time of around 34 min, with a work-to-rest ratio of 5:2. They are also required to perform approximately 100 high-intensity exercises lasting 7 to 14 s each, with these exercises cumulatively representing about half of the entire match play time. Additionally, water polo athletes engage in sprint swimming efforts, covering an average of 60 swims per game, with each swim lasting from 10 to 12 s. This amounts to a total swimming distance of 500 to 800 m, with athletes spending up to 45–55% of match time in a horizontal position. As a result, water polo can be characterized as both an aerobic and anaerobic sport, demanding significant energy expenditure [10,26,33]. The higher energy intake observed in swimmers (56.2 to 61.85 kcal/kg) compared to water polo athletes (35.4 to 42.0 kcal/kg) aligns with the greater energy demands of swimming due to the higher training volume and intensity involved in the sport, and in our study swimmers had more training volume and intensity than water polo athletes. However, it is noteworthy that previous cross-sectional studies have reported low energy intake among swimmers [34,35], and longitudinal studies have found similar results, indicating that some swimmers may have low energy intakes even at the elite level [36]. This discrepancy may be attributed to individual differences in dietary practices, training loads, and metabolic rates among athletes. 

The higher protein intake observed in swimmers compared to WP athletes is consistent with findings from previous studies in elite swimmers [12,37]. Swimmers often require higher protein intake to support muscle repair, recovery, and adaptation, especially if they are attempting to lose weight while minimizing muscle loss [38]. In our study, water polo athletes have intakes less than swimmers, but this value was insufficient to create or maintain SMM. Additionally, to promote muscle repair and remodeling and improve post-exercise strength- and hypertrophy-related responses, it is crucial to consume PRO before, during, and after a workout [39]. Intake of PRO during these periods has been associated with a favorable impact on muscle protein synthesis (MPS) [40]. Stokes et al. [41] expressed that for the promotion of muscle hypertrophy and strength, 1.6–2.2 g/kg/day of PRO is required, and Vitale et al. [42] admitted that for endurance athletes, the optimal limit is between 1.2 and 2.0 g/kg/day for the improvement of performance and recovery.

In general, athletes are advised to adopt specific CHO intake strategies to optimize their performance. In the 48 h leading up to a competition, it is recommended that athletes consume approximately 10–12 g of CHO per kilogram of body weight per day, a quantity similar to what is advised prior to high-intensity interval sessions [6,43,44]. Additionally, 4 h before the competition, the recommended CHO intake should be in the range of 1–4 g/kg. Interestingly, our study revealed that both swimmers and WP athletes had lower daily CHO intake values compared to the recommended levels despite the fact that swimmers had higher overall macronutrient intake than water polo athletes. This discrepancy in CHO intake might be influenced by the athletes’ proximity to an important international competition. Athletes often engage in a tapering phase before competitions, wherein they reduce the intensity and volume of their training to enhance their athletic performance. This tapering phase is frequently accompanied by a reduction in nutritional intake, which could explain the lower CHO consumption observed in our study. During the tapering phase, it is crucial for athletes to pay close attention to their energy intake to prevent energy imbalances and undesirable changes in body composition [45]. While reducing energy intake during tapering, athletes should ensure that their intake of micronutrients and water remains sufficient. Therefore, adequate carbohydrate intake can reduce the incidence of exercise-induced hypoglycemia. Muscle glycogen depletion depends on the duration and intensity of exercise, suggesting that the recommended daily carbohydrate intake for elite male athletes is 8–12 g/kg/day for optimal replenishment of glycogen stores [46,47]. In a comparative study conducted by Farajian et al. [38], the dietary intake of 31 Greek national swimmers and 27 water polo athletes was examined. According to dietary recall and FFQ, surprisingly no significant differences were observed in terms of energy or macronutrient intake between the athletes involved in the two distinct aquatic sports disciplines despite the WP athletes showing a lower CHO intake, similar to the findings of our current study. The importance of CHO intake in athletic performance warrants special attention. A study demonstrated that a reduction of 10% in daily CHO intake had an effect on the performance of swimmers in the 365 m freestyle test. Conversely, increasing CHO intake by 10% resulted in notable improvements in both the 91.5 and 365 m tests [43,47]. These findings emphasize the critical role of CHO in optimizing athletic performance, particularly in endurance-based activities like swimming. Furthermore, it is worth noting that athletes often face challenges in maintaining adequate CHO intake during competitions. Consequently, there is a recommendation for athletes to engage in “super-compensation” CHO intake strategies [43,48], wherein they strategically increase their CHO consumption to ensure sufficient glycogen stores and support optimal performance during events. However, discrepancies in outcomes between different studies can be attributed to various factors. The variability in results may be influenced by aspects such as sample size, participant characteristics, methodological differences, and potential confounding variables. Therefore, researchers and practitioners must consider these factors when interpreting and comparing findings from diverse studies within the field of sports nutrition. Acknowledging these nuances is essential for establishing a comprehensive understanding of the relationship between CHO intake and athletic performance in aquatic sports and beyond. Also, fat intake observed in this study aligns with previous research [34,35,36], indicating that both swimmers and WP athletes have appropriate fat intake levels, which is essential for various physiological functions and energy storage. Finally, further research encompassing larger sample sizes and meticulous experimental design may aid in refining our comprehension of the intricate interplay between dietary CHO intake and athletic performance.

Physical activity patterns and dietary behaviors are closely interlinked and determine body composition and health outcomes. Domaradzki et al. and demonstrated unhealthy dietary triads and minimal physical activity were prevalent among late adolescents and demonstrates sex differences in healthy and unhealthy behaviors; females had a stronger relationship between dietary behaviors and body composition [49]. Similarly, Pellegrini et al. [50] noted significant weight gain in adults with obesity during the COVID-19 lockdown, attributed to decreased physical activity and increased consumption of unhealthy foods, exacerbated by psychological stressors such as anxiety and depression. This is further supported by Domaradzki et al. [51], who identified specific physical activity and dietary behaviors that discriminate between different body fat groups, suggesting tailored interventions can enhance healthy habits. To this end, Godala et al. [52] demonstrated healthy dietary practices and regular physical activity significantly reduce the risk of metabolic syndrome. Lastly, a longitudinal study by Lake et al. [53] demonstrates that dietary habits established in adolescence significantly influence dietary patterns in adulthood and that socio-economic status, gender, and location can impact dietary behaviors. Collectively, these findings align with our study, which emphasizes targeted nutritional interventions for elite athletes, where distinct physical activity patterns and dietary behaviors are present among swimmers and water polo players, highlighting the need for customized nutritional strategies to optimize performance.

The limitations of the current study are indeed important to consider for a comprehensive understanding of the research outcomes. Some of the key limitations include the following: (1) The study included only male athletes and therefore did not investigate potential sex-specific differences in body composition and dietary intake. (2) Position-specific differences: the study did not assess position-specific physiological, physical, and nutritional demands within the same sport or distance in swimmers. (3) Timing of data collection: the data collection took place 8 weeks before an international competition, which may not fully capture the variations in energy demands and intake that could occur throughout an entire season or Olympic cycle. (4) Generalizability: the study focused on elite athletes, and the findings may not be applicable to swimmers and water polo athletes at lower competition levels or with different training backgrounds. Considering these limitations, the findings can still serve as a valuable reference and provide useful insights for coaches and sports practitioners working with elite male swimmers and water polo athletes. 

Future investigations should explore how the specific positions or roles within a team might impact body composition and dietary requirements and should include larger and more diverse athlete populations. Longitudinal studies and randomized controlled trials can offer valuable insights into causal relationships with athletic performance. Thus, future research should incorporate these considerations for a more comprehensive understanding of the factors impacting body composition, nutrition, and performance in aquatic sports.

## 5. Conclusions

Comparative analysis of water polo athletes showed significantly greater height, BMI, FFM, SMM, and overall weight compared to swimmers. In contrast, swimmers demonstrated higher mean daily energy and macronutrient intake, indicating a potentially elevated caloric demand associated with their training regimen. Water polo athletes may benefit from programs designed to maximize their physical stature and strength, capitalizing on their unique physiological profile. Swimmers, on the other hand, might focus on dietary strategies to sustain their elevated energy expenditure, ensuring optimal performance in the pool. Ultimately, these insights provide valuable guidance for coaches, trainers, and nutritionists working with swimming and water polo athletes, emphasizing the need for individualized nutritional approaches to meet the specific demands of each sport.

## Figures and Tables

**Table 1:** Age and morphological characteristics of swimmers and WP athletes. BMI: Body Mass Index; SMM: Skeletal Muscle Mass; Extremely Large: EL; Water Polo: WP.

	Swimmers *n* = 10	WP Athletes *n* = 13	Comparison t; *p*	Effect Size (Hedges’ g)	95% Confidence Interval	
Age (years)	22.1 ± 2.2 [21.0 to 24.3]	24.4 ± 1.8 [23.6 to 26.2]	−2.77; 0.29	1.12 Moderate	0.25	1.97
Height (cm)	186.5 ± 2.0 [185.0 to 188.0]	189.4 ± 2.9 [187.7 to 191.2]	−2.70; 0.013	1.13 Moderate	0.22	1.94
Body weight (kg)	76.7 ± 2.2 [75.1 to 78.4]	86.9 ± 6.9 [82.7 to 91.1]	−4.98; <0.001	1.88 Large	0.83	2.76
BMI (kg∙m^−2^)	22.1 ± 0.55 [21.8 to 22.6]	24.3 ± 1.4 [23.4 to 25.1]	−4.8; <0.001	1.96 Large	0.82	2.73
Body fat (%)	11.3 ± 0.43 [11.0 to 11.6]	7.8 ± 0.63 [7.4 to 8.2]	15.0; <0.001	6.32 EL	4.08	8.06
Fat mass (kg)	8.7 ± 0.4 [8.4 to 9.0]	6.8 ± 0.4 [6.5 to 7.1]	10.3; <0.001	4.75 EL	2.69	5.68
SMM (kg)	43.9 ± 1.65 [42.7 to 45.1]	47.1 ± 1.32 [46.3 to 47.9]	−5.22; <0.001	2.17 Very large	1.09	3.13
Fat-free mass (kg)	61.1 ± 1.38 [60.1 to 62.1]	62.9 ± 1.4 [62.0 to 63.7]	−2.90; 0.008	1.29 Large	0.31	2.06

**Table 2:** Daily micronutrient intake/kg of swimmers and water polo athletes. Fe: Iron; Ca^+^: Calcium; Se: Selenium; P: Phosphorus; Na^+^: Sodium; K^+^: Potassium; Data are mean ± SD [95% confidence intervals]. Extremely large: EL; WP: Water Polo.

	Swimmers *n* = 10	WP Athletes *n* = 13	Comparison t; *p*	Effect Size (Hedges’ g)	95% Confidence Interval	
Vit A (mg/kg)	24.5 ± 4.6 [21.1 to 27.8]	17.7 ± 3.7 [15.5 to 20.2]	3.85; 0.001	1.65 Moderate	0.002	1.67
Vit B1 (mg/kg)	0.022 ± 0.002 [0.021 to 0.024]	0.023 ± 0.007 [0.019 to 0.028]	−0.36; 0.71	0.73 Moderate	−0.29	1.33
Vit B2 (mg/kg)	0.032 ± 0.007 [0.026 to 0.037]	0.0314 ± 0.011 [0.024 to 0.038]	0.15; 0.87	0.10 Trivial	−0.53	1.07
Vit B3 (mg/kg)	0.039 ± 0.013 [0.030 to 0.048]	0.129 ± 0.029 [0.111 to 0.147]	−8.93; <0.001	3.64 Very large	2.15	4.78
Vit B6 (mg/kg)	0.30 ± 0.03 [0.28 to 0.32]	0.24 ± 0.03 [0.22 to 0.26]	4.05; 0.01	0.63 Very large	−0.19	1.44
Vit B9 (mcg/kg)	2.47 ± 0.40 [2.18 to 2.76]	2.17 ± 0.36 [1.95 to 2.39]	1.86; 0.076	0.79 Moderate	−0.77	0.82
Vit B12 (mcg/kg)	0.10 ± 0.01 [0.09 to 0.11]	0.13 ± 0.02 [0.11 to 0.14]	−3.11; 0.005	1.82 Large	0.67	2.54
Vit C (mg/kg)	1.03 ± 0.11 [0.95 to 1.12]	0.59 ± 0.16 [0.49 to 0.69]	7.12; <0.001	1.95 Very large	0.94	2.92
Fe (mg/kg)	0.42 ± 0.06 [0.37 to 0.47]	0.36 ± 0.03 [0.33 to 0.38]	9.90; 0.008	0.25 Large	−0.55	1.05
Se (mcg/kg)	0.33 ± 0.04 [0.30 to 0.36]	0.64 ± 0.04 [0.58 to 0.70]	−9.16; <0.001	4.25 EL	2.85	5.94
Ca^+^ (mg/kg)	17.7 ± 1.9 [16.4 to 19.1]	14.3 ± 2.4 [12.9 to 15.8]	3.68; 0.001	0.58 Large	−0.24	1.39
P (mg/kg)	15.0 ± 2.8 [14.4 to 15.6]	23.7 ± 2.8 [21.9 to 25.4]	−9.36; <0.001	3.98 Very large	2.29	5.01
Na^+^ (mg/kg)	68.7 ± 6.2 [64.2 to 73.2]	74.2 ± 8.5 [68.9 to 79.1]	−1.65; 0.12	1.95 Very large	0.95	2.92
K^+^ (mg/kg)	37.1 ± 2.6 [35.2 to 39.0]	35.0 ± 4.3 [36.3 to 37.6]	1.39; 0.17	0.59 Small	0.03	1.63

## Data Availability

Data and statistical analyses are available upon request on a case-by-case basis for non-commercial scientific inquiry and educational use if IRB restrictions and research agreement terms are not violated.
